# Hemophagocytic Lymphohistiocytosis due to Brucellosis in a Xeroderma Pigmentosum Pediatric Patient: A Case Report and Review of the Literature

**DOI:** 10.1155/crh/8931263

**Published:** 2025-10-21

**Authors:** Reem Shihab, Sultan Mosleh, Muhammad Takhman, Fadi Yousef, Marian Salim, Asala Abuabed, Sara Abueisheh, Mohammad Abed

**Affiliations:** ^1^Department of Medicine, Faculty of Medicine and Health Sciences, An-Najah National University, Nablus, State of Palestine; ^2^Department of Pediatrics, An-Najah National University, Nablus, State of Palestine

## Abstract

We report a pediatric patient with xeroderma pigmentosum (XP) who developed hemophagocytic lymphohistiocytosis (HLH) secondary to Brucella infection—an exceedingly rare occurrence. XP is a rare autosomal recessive genetic disorder characterized by extreme ultraviolet radiation (UVR) sensitivity due to the inability to repair DNA pyrimidine dimers caused by UV exposure. This defect leads to a markedly increased risk of skin cancer and progressive neurological degeneration (Leung, 2022). HLH is a rare, potentially fatal hypersensitivity syndrome characterized by excessive activation and impaired downregulation of T-lymphocytes and macrophages. This dysregulation results in an overproduction of proinflammatory cytokines, destruction of blood cells, and subsequent tissue and organ damage (Fisman, 2000). While secondary HLH may follow various infections, Brucella-induced HLH is rare (Wolska, 2006), and to our knowledge, this is the first reported case in a patient with XP.

## 1. Introduction

In this report, we present an unusual case of an 8 year-old female patient with XP, who exhibited prolonged and recurrent fever, abdominal and back pain, arthralgia, splenomegaly (15 cm), and pancytopenia. She was diagnosed with secondary HLH triggered by brucellosis, an association rarely reported in the literature. Early recognition of Brucella as a risk of HLH, especially in patients with underlying genetic diseases, can guide physicians to detect the condition as soon as possible and initiate life-saving interventions.

Xeroderma pigmentosum (XP) is an autosomal recessive genetic disorder characterized by deficient DNA repair mechanisms, leading to DNA damage upon ultraviolet radiation (UVR) exposure [1]. It has a variable incidence around the world, ranging from (1–45) per million live births, with a higher incidence in countries where consanguineous marriages are common [1]. Eight genetic mutations have been identified as a causative factor for XP. The first seven (XPA, XPB, XPC, XPD, XPE, XPG, and XPV) are responsible for nucleotide excision repair (NER) that is required in UVR-induced photoproduct repair, while XPV mutation causes a defect in replicating DNA with UVR damage [2].

It is characterized by photosensitivity, freckle-like pigmentations on all exposed skin, which usually starts before 2 years of age, and high susceptibility to skin cancers. Twenty-five percent of patients also have neurological manifestations (e.g., microcephaly, sensorineural hearing loss, diminished or absent deep tendon stretch reflexes, cognitive impairment, and ataxia) [1].

Treatment for XP is supportive and depends on the affected organs, where skin lesions can be treated with freezing, topical agents (e.g., imiquimod and fluorouracil), oral agents (e.g., isotretinoin), or surgery for high-risk cases; eye neoplasms are managed surgically; and hearing loss can be improved with hearing aids. Preventive measures include avoiding UV exposure, which is aided by monitoring environments with UV light meters. Regular surveillance involves skin, eye, neurological, and hearing exams. Their life expectancy is 29 years in those with neurodegeneration and 37 years in those without [1].

Hemophagocytic lymphohistiocytosis (HLH) is a rare, frequently fatal hypersensitivity syndrome characterized by excessive activation and impaired downregulation of T-lymphocytes and macrophages, which leads to excessive proinflammatory cytokines release, destruction of blood cells, and tissue and organ damage [3]. It is estimated that HLH incidence is 1-2 per 1 million of the general population annually [4]. However, HLH is more common in the pediatric population, with an incidence of one per 50,000 children [5]. Diagnosis is based on HLH-2004 criteria [6].

HLH can be divided into primary (familial) HLH and secondary HLH. Primary HLH typically appears in early childhood and is often caused by mutations that affect cytotoxic cell function. Meanwhile, secondary HLH arises at any age due to external triggers that cause immune vulnerability, such as autoimmune diseases, malignancies, and infections. EBV is the most common infectious cause [7]. Other causes of HLH include cytomegalovirus, parvovirus B19, human immunodeficiency virus (HIV), and human herpesvirus-6 [8].

Treatment of HLH focuses on reducing hypercytokinemia and eliminating activated and infected cells. Treatment options for primary HLH include immunosuppressive, immunomodulatory, and cytostatic drugs; T-cell antibodies; agents targeting cytokines; and hematopoietic stem cell transplantation (HSCT) to suppress immunity [8]. Secondary HLH treatment aims to treat the underlying cause, along with the standard HLH treatment of chemotherapy and immunomodulatory agents [9]. G-CSF and blood products may be considered [8].

One rare cause of secondary HLH is Brucella bacteria, an intracellular infectious agent acquired by consuming unpasteurized dairy products, direct contact with infected animals, and inhalation. The bacteria can cause brucellosis, a chronic granulomatous disease requiring prolonged and combined antibiotic treatment [9].

As the most common zoonotic infection [10], Brucellosis usually presents with various constitutional symptoms (e.g., fever, malaise, and arthralgias), hepatomegaly, splenomegaly, and lymphadenopathy [9].

However, in rare cases, especially in regions with higher exposure to Brucella, it can lead to HLH [11].

## 2. Case Presentation

**Figure 1:**
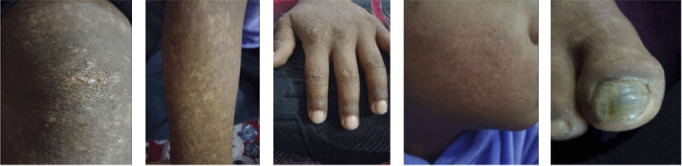
Hypopigmented and hyperpigmented macules over the knees (a), forearm (b), dorsum of the hand (c), and face (d). (e) Actinic keratosis of the nail with bluish pigmentation.

An 8-year-old Palestinian female patient, who was diagnosed with XP diagnosed within the first few months of life, presented with gross motor developmental delay and speech impairment. She began walking at 1.5 years of age and spoke her first two words by the age of one. She exhibits the typical cutaneous manifestations, including a diffuse skin rash with patches of discolored skin resembling severe aging, as shown in Figure 1. Her family history is notable for parental consanguinity and a brother with XP and intellectual disability.

In April 2024, the patient presented to a Jenin Governmental Hospital with a 3-day history of fever, generalized fatigue, abdominal pain, and arthralgia. Initial labs revealed pancytopenia, and Brucella serology was positive. She was hospitalized for 10 days. However, due to a lack of documentation in her medical records, the specific treatment plan administered during that admission is unknown. She was subsequently referred to An-Najah National University Hospital (NNUH) for bone marrow biopsy evaluation, which showed normocellular trilineage hematopoiesis with evidence of hemophagocytosis. She was discharged on a 6-week course of oral doxycycline and rifampin.

In July of 2024, the patient experienced recurrent episodes of fevers spiking up to 38°C managed with antipyretics by the parents. As the episodes persisted, further evaluation was undertaken. On July 27^th,^ 2024, an abdominal ultrasound showed splenomegaly (15 cm) and a right inguinal lymph node enlargement. On the fifth of August, the patient presented with fever, arthralgia, back pain, decreased appetite, and hypoactivity. Brucella testing was again positive. Laboratory results (Table 1) demonstrated persistent pancytopenia, elevated inflammatory markers (C-reactive protein and erythrocyte sedimentation rate), hyperferritinemia, and elevated triglycerides and lactate dehydrogenase (LDH), raising concerns for HLH. This prompted a second bone marrow biopsy.

**Figure 2:**
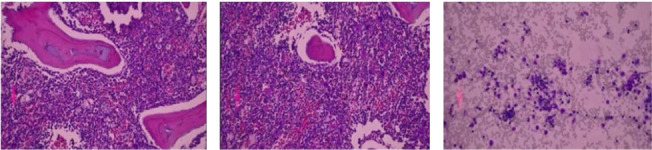
Bone marrow aspirate showing evidence of hemophagocytosis, where macrophages engulf blood cells and contain cellular debris, using (a) hematoxylin, (b) eosin stain, and (c) wright-giemsa stain.

Bone marrow aspirate findings confirmed the presence of hemophagocytes (Figure 2), supporting the HLH diagnosis. The patient was treated with rifampin (400 mg once daily for 6 weeks), doxycycline (50 mg twice daily for 6 weeks), and piperacillin/tazobactam (2000 mg IV). She responded well to treatment, as evidenced by normalization of her lab results and improvement in her symptoms.

## 3. Discussion

Brucellosis-induced HLH is rare, with limited cases reported in the literature. We conducted a comprehensive literature review using PubMed, Google Scholar, and other relevant databases but were unable to identify any previously reported cases of brucellosis-induced HLH in patients with XP. To the best of our knowledge, this is the first reported case. Our PubMed search using the terms “Brucella OR brucellosis” AND “HLH OR hemophagocytic lymphohistiocytosis” identified 18 case reports published between 2006 and 2024, describing 21 patients [12].

A prior literature review of 22 patients reported fever in all cases, splenomegaly in 7, hemoglobin levels below 9 g/dL in 7, neutrophil counts under 1 × 10^3^/μL in 13, platelet counts below 100 × 10^9^/L in 20, triglycerides above 265 mg/dL in 9, ferritin ≥ 500 ng/mL in 15, and fibrinogen levels under 150 mg/dL in 7. Blood cultures were positive in 14 patients, and bone marrow examination revealed hemophagocytosis in 16. These laboratory findings are consistent with our case, except for the platelet count (108 × 10^9^/L) and fibrinogen level (365 mg/dL). Soluble IL-2 receptor levels and NK cell activity were not investigated in most of the patients in the reviewed literature, nor in our patient.

Seventeen of the reviewed cases were treated with antibiotics alone, with complete recovery in 16, with a similar outcome to our patients. It is worth noting that in some reports, it was unclear whether certain laboratory tests had been performed. Comparison between our case and the published cases indicates strong parallels in both treatment approaches and outcomes [13]. This suggests that in infection-driven HLH, pathogen eradication can be sufficient, though immunosuppressive therapy may be necessary in severe or refractory cases [13].

The coexistence of these conditions carries important clinical implications. In XP, ultraviolet (UV) exposure triggers immune overactivation with elevated levels of interleukin-1 (IL-1), interleukin-6 (IL-6), and interferon-gamma (IFN-γ)—cytokines that also play central roles in HLH pathogenesis. This shared cytokine profile, with the addition of the fact that Brucella disrupts immune regulation via interleukin-10 (IL-10), a cytokine with potent anti-inflammatory effects. For this reason, the condition often requires the use of both antibiotics and immunosuppressants to prevent deterioration [8]. These immunopathological mechanisms also help explain why patients with XP are more predisposed to exaggerated inflammatory responses, thereby heightening the risk of HLH during infectious episodes.

Brucella's ability to persist within macrophages prolongs immune activation, driving cytokine overproduction (IFN-γ, TNF-α, and IL-6) and creating a “cytokine storm.” Immune dysregulation in XP further exacerbates this process. Defective macrophage bactericidal function, impaired dendritic cell antigen presentation, diminished NK cell cytotoxicity, and compromised T-cell responses collectively reduce infection control and prolong inflammation. This immune deficit likely contributed to our patient's recurrent fevers and prolonged hyperinflammatory state [14, 15].

Understanding the interaction between XP-related immune dysfunction and intracellular pathogens such as Brucella is essential. It suggests that XP patients in endemic regions may warrant heightened surveillance for HLH when presenting with systemic infections, and that clinicians should consider Brucella testing early in the diagnostic process.

## 4. Conclusion

This case represents the first reported case of brucellosis-induced HLH in a patient with XP. As a singular report, the findings cannot be generalized; the overlap in immune dysregulation between XP and HLH suggests a potential predisposition to severe infection-related hyperinflammation. Further studies and case series are needed to elucidate the relationship between XP, HLH, and brucellosis. Clinicians should maintain a high index of suspicion for HLH in XP patients presenting with systemic infection, particularly in Brucella-endemic areas. Long-term follow-up of such cases would provide critical insights into disease progression and treatment outcomes, particularly in XP patients.

## Figures and Tables

**Table 1:** Lab results on 11^th^ of August 2024 showing pancytopenia, elevated inflammatory markers (CRP and ESR), and hyperferritinemia, with elevated triglycerides and LDH.

Test	Value	Normal range	Notes
WBC	1.58 × 10^9^/L	4.5–11 × 10^9^/L	Low
Hemoglobin	8.1 g/dL	11.5–15.5 g/dL	Low
MCV	62.3 fL	80–100 fL	Low
Platelets	108 × 10^9^/L	150–450 × 10^9^/L	Low
ANC	260	> 1500	Low
Fibrinogen	365 mg/dL	200–400 mg/dL	Normal
Ferritin	2786 ng/mL	7–140 ng/mL	High
LDH	1155 U/L	140–280 U/L	High
Triglycerides	283 mg/dL	< 150 mg/dL	High
ESR	45 mm/hr	< 20 mm/hr	High
Reticulocyte absolute count	91.6 × 10^9^/L	25–75 × 10^9^/L	High
Reticulocyte count %	2.35%	0.5%–1.5%	High
Immature retic fraction	22.7%	< 5%	High
Albumin	3.5 g/dL	3.4–5.4 g/dL	Normal
Total bilirubin	0.287 mg/dL	0.1–1.2 mg/dL	Normal
Direct bilirubin	0.129 mg/dL	0.0–0.4 mg/dL	Normal
CRP	28.7 mg/L	< 5 mg/L	High
Alkaline phosphatase	102 U/L	44–147 U/L	Normal
Calcium	8.7 mg/dL	8.5–10.5 mg/dL	Normal
Magnesium	2.4 mg/dL	1.7–2.2 mg/dL	High
Sodium	136.7 mmol/L	135–145 mmol/L	Normal
Potassium	4.3 mmol/L	3.5–5.1 mmol/L	Normal
Chloride	102 mmol/L	98–106 mmol/L	Normal

## Data Availability

No datasets were generated or analyzed for the current study.
